# The Role of NLRP3 Inflammasome in Type 2 Diabetes Mellitus and Its Macrovascular Complications

**DOI:** 10.3390/jcm14134606

**Published:** 2025-06-29

**Authors:** Konstantinos Karamitsos, Evangelos Oikonomou, Panagiotis Theofilis, Ignatios Ikonomidis, Eva Kassi, Vaia Lambadiari, Elias Gialafos, Aikaterini Tsatsaragkou, Vasiliki-Chara Mystakidi, Konstantinos Zisimos, Kyriakos Dimitriadis, Dimitris Tousoulis, Gerasimos Siasos

**Affiliations:** 1 3rd Department of Cardiology, Sotiria Chest Disease Hospital, Medical School, National and Kapodistrian University of Athens, 11527 Athens, Greece; kwnkaramitsos@gmail.com (K.K.); gialaf@yahoo.com (E.G.); aiktsatsaragou@hotmail.com (A.T.); xaram25@gmail.com (V.-C.M.); zisimoskostas@gmail.com (K.Z.); ger_sias@hotmail.com (G.S.); 2 1st Department of Cardiology, Hippokration General Hospital, Medical School, National and Kapodistrian University of Athens, 11527 Athens, Greece; panos.theofilis@hotmail.com (P.T.); dimitriadiskyr@yahoo.gr (K.D.); drtousoulis@hotmail.com (D.T.); 3 2nd Cardiology Department, Attikon University Hospital, National and Kapodistrian University of Athens, 12462 Athens, Greece; ignoik@gmail.com; 4 Department of Biological Chemistry, Medical School, National and Kapodistrian University of Athens, 11527 Athens, Greece; evakassis@gmail.com; 5 2nd Department of Internal Medicine, Attikon University Hospital, Medical School, National and Kapodistrian University of Athens, 12462 Athens, Greece; vlambad@otenet.gr

**Keywords:** diabetes mellitus, inflammation, NLRP3 inflammasome

## Abstract

Diabetes Mellitus (DM) is among the most common non-infectious causes of death globally, with Type 2 DM (T2DM) representing the majority of cases. T2DM is primarily characterized by insulin resistance, leading to hyperglycemia and compensatory hyperinsulinemia. Rapid changes in lifestyle, technological advancement, and societal evolution have fueled a global rise in T2DM, making it a major public health concern. The condition is associated with numerous complications—both macrovascular and microvascular—including coronary artery disease, heart failure, chronic kidney disease, and diabetic retinopathy, all of which contribute to increased morbidity and early mortality. Chronic tissue inflammation is now recognized as a key factor in the development of T2DM, with elevated inflammatory markers serving as predictors of the disease. In particular, the NLRP3 inflammasome complex has emerged as a central player in this inflammatory process. NLRP3 acts as an intracellular sensor for danger signals and tissue injury, triggering inflammatory responses and contributing to endothelial dysfunction and T2DM pathogenesis. Its role in linking metabolic stress to inflammation has positioned it as a promising therapeutic target. This review focuses on the mechanisms underlying NLRP3 inflammasome activation and its role in T2DM and related vascular complications. Additionally, it highlights emerging therapies that target NLRP3, offering new potential strategies for the prevention and treatment of T2DM.

## 1. Introduction

Diabetes Mellitus (DM) is a chronic endocrine disorder marked by elevated plasma glucose levels, and it is projected to affect around 700 million adults by 2045 [1]. Type 2 Diabetes Mellitus (T2DM), accounting for 90% of cases, arises from a combination of genetic and environmental factors, including obesity, physical inactivity, smoking, and alcohol use [2]. T2DM is associated with serious macrovascular and microvascular complications—such as coronary artery disease, heart failure, stroke, peripheral artery disease (PAD), retinopathy, nephropathy, and neuropathy—which contribute to increased morbidity and reduced quality of life [3,4].

Emerging evidence identifies chronic, low-grade systemic inflammation as a key contributor to both the onset and progression of T2DM and its complications. While the exact mechanisms remain under investigation, hyperglycemia, insulin resistance, and excess fatty acids promote oxidative stress, disrupt cellular signaling, and lead to vascular inflammation and thrombosis [1,2,5,6,7].

Recent studies have focused on the role of inflammasomes—intracellular multi-protein complexes that regulate inflammation [2]—in the pathogenesis of diabetic complications. The NOD-like receptor family, pyrin domain-containing 3 (NLRP3) inflammasome in particular, has been shown to activate caspase-1, leading to the release of the pro-inflammatory cytokines interleukin (IL)-1β and IL-18 and to inducing pyroptosis. IL-1β plays a critical role in the vascular damage seen in DM [8].

This review explores the activation and regulatory mechanisms of inflammasomes in T2DM, particularly their role in macrovascular complications. It also highlights the therapeutic potential of targeting the NLRP3 inflammasome as a novel strategy for managing T2DM and its associated vascular outcomes.

## 2. Search Methodology

This article is designed as a narrative review, aiming to provide a comprehensive and integrative overview of the current knowledge regarding the NLRP3 inflammasome and its role in T2DM and related macrovascular complications. The relevant literature was identified through searches in PubMed Scopus, Google Scholar, and Web of Science using combinations of keywords, including “NLRP3 inflammasome”, “type 2 diabetes mellitus”, “macrovascular complications”, “cardiovascular disease”, and “inflammation”, among others. Studies were selected based on relevance, recency (with preference for publications within the past 10 years), and their contribution to understanding molecular mechanisms, clinical correlations, or therapeutic implications. Both preclinical and clinical studies were considered, and references were cross-checked to ensure the comprehensive coverage of the topic.

## 3. The NLRP3 Inflammasome; Structure and Activation

Inflammasomes are intracellular multiprotein complexes that act as part of the innate immune system, coordinating inflammatory responses to microbial and non-microbial stimuli [9]. First identified in 2002, these cytosolic structures typically consist of three components: a pattern recognition receptor (PRR), the adaptor protein ASC (which contains PYD and CARD domains), and the inflammatory protease pro-caspase-1 [10]. Upon activation, this complex promotes the maturation of pro-inflammatory cytokines IL-1β and IL-18, as well as inducing a form of programmed cell death known as pyroptosis [11,12,13,14].

Among inflammasomes, NLRP3 is the most extensively studied due to its role in various diseases, including DM, atherosclerosis, gout, and neurodegeneration. Structurally, the NLRP3 protein consists of a C-terminal leucine-rich repeat (LRR) domain that detects danger signals, a central NACHT domain responsible for oligomerization, and an N-terminal PYD domain for interaction with ASC [12,15,16].

Canonical activation follows a two-step process: priming and activation [13]. Priming is initiated by Toll-like receptors (TLRs), cytokines like tumor necrosis factor (TNF)-α, or NOD-like receptors detecting pathogen- or damage-associated molecular patterns (PAMPs/DAMPs) [13,16,17,18]. This triggers NF-κB signaling, which upregulates the expression of NLRP3, pro-IL-1β, and pro-IL-18 [13,16,17,18]. Post-translational modifications, such as ASC phosphorylation and NLRP3 deubiquitination, also modulate the process [19]. Activation involves a second signal that induces conformational changes in NLRP3, leading to its oligomerization. This recruits ASC via PYD-PYD interaction and then pro-caspase-1 through CARD-CARD interaction, leading to caspase-1 activation and cytokine processing [16].

Several intracellular signals are proposed as triggers of NLRP3 activation. Ion flux, particularly K^+^ efflux, is one of the earliest recognized signals [13]. ATP binding to P2X7 receptors induces a K^+^ efflux, facilitating NLRP3 activation [14,19]. Calcium (Ca^2+^) and chloride (Cl^−^) fluxes have also been implicated, although their exact roles remain under investigation [18]. Reactive oxygen species (ROS), generated in response to various stimuli, are believed to act upstream of NLRP3 activation [19]. ROS facilitate NLRP3 assembly via the thioredoxin-interacting protein (TXNIP), which binds to NLRP3 in oxidative conditions [10,14,17,19,20]. Lysosomal membrane rupture occurs when phagocytosed crystalline or particulate matter causes lysosomal damage [14]. This leads to the release of cathepsin B into the cytosol, which promotes NLRP3 activation [10,12,14,17,18,19]. Mitochondrial dysfunction contributes to activation via mitochondrial ROS (mtROS), mitochondrial DNA (mtDNA), and mitochondrial lipids like cardiolipin, which may serve as docking or activating molecules [12,17,21].

The non-canonical pathway involves the intracellular detection of bacterial lipopolysaccharide (LPS) by caspase-4 and -5 in humans (caspase-11 in mice) [9,15,18]. These caspases directly bind LPS, leading to their activation and cleavage of gasdermin D (GSDMD), which forms pores in the plasma membrane, initiating pyroptosis [9,15,18]. Pore formation allows for ATP release, which then activates the canonical NLRP3 pathway through P2X7 receptors and subsequent K^+^ efflux [9,12,13,16,17,18]. Hence, the non-canonical pathway contributes both to pyroptosis and canonical inflammasome activation [15].

In the alternative pathway, human monocytes stimulated by LPS can activate caspase-1 and produce IL-1β without the need for a second signal or K^+^ efflux [12,13,17]. This process involves caspase-8, activated through the TLR4–TRIF–RIPK1–FADD–caspase-8 axis [12,13,17]. Notably, this pathway does not induce pyroptosis or require ASC speck formation and reflects crosstalk between apoptosis and inflammasome signaling [12].

While the NLRP3 inflammasome is among the most extensively studied, it does not act in isolation. Recent research has revealed that multiple inflammasome sensors—including NLRP1, NLRC4, and AIM2—can be co-activated under certain conditions and may converge on shared signaling hubs. This convergence can lead to a form of inflammatory cell death termed PANoptosis, which is a coordinated interplay of pyroptosis, apoptosis, and necroptosis [22]. PANoptosis is driven by the PANoptosome, a multimeric complex that integrates signals from sensors such as Z-DNA-binding protein 1 and components of multiple inflammasomes, thereby enhancing the inflammatory response [23]. PAMPs and DAMPs, both mentioned earlier as triggers of NLRP3, can similarly stimulate other inflammasomes, suggesting a synergistic and sometimes redundant network of inflammatory control.

## 4. Regulation of T2DM by NLRP3 Inflammasome

The NLRP3 inflammasome acts as a key intracellular sensor of metabolic danger signals, responding to various endogenous and exogenous stimuli that accumulate during obesity and contribute to chronic low-grade inflammation (Table 1). These activators include saturated fatty acids, ceramides, elevated glucose, uric acid, and islet amyloid polypeptide. Its activation, and the resulting production of IL-1β, were first observed in pancreatic β-cells and macrophages infiltrating the islets, triggering local inflammation and amplifying the release of cytokines and chemokines [19,24]. This process has been increasingly recognized as a central driver of obesity-related disorders, particularly T2DM.

Inflammation arising from nutrient overload involves both innate and adaptive immune responses, with PRR on adipocytes and immune cells detecting nutrient-derived danger signals. These interactions activate pro-inflammatory signaling pathways and contribute to the development of systemic insulin resistance.

Studies in both humans and animal models consistently show a strong correlation between obesity, insulin resistance, and increased NLRP3 expression in adipose tissue [19,33]. High-fat diets (HFDs) significantly upregulate NLRP3 in mice, whereas caloric restriction reduces its expression. The inhibition or genetic deletion of NLRP3 confers protection against diet-induced insulin resistance and obesity [34]. Similarly, in individuals with T2DM, lifestyle interventions, such as caloric restriction and exercise, reduce NLRP3 expression, emphasizing its role as a modifiable inflammatory target [19,35].

In adipose tissue, infiltrating pro-inflammatory macrophages secrete cytokines, including TNF, IL-1β, and IL-6, promoting insulin resistance. Moreover, NLRP3 activation influences adipocyte differentiation, favoring the emergence of insulin-resistant phenotypes.

Plasma free fatty acids, especially those elevated by high-fat dietary intake, play a critical role in activating the NLRP3 inflammasome. Among these, palmitic acid has been shown to promote mitochondrial ROS production and lysosomal destabilization, both of which contribute to NLRP3 activation and insulin resistance [19]. Palmitate also disrupts endothelial tight junctions, linking metabolic dysfunction to vascular damage. In intestinal epithelial cells, a high-cholesterol diet induces IL-1β-mediated myeloid cell infiltration and caspase-1 activation, further contributing to systemic inflammation [36]. Additionally, palmitate activates caspase-4/5 in monocytes, driving the release of IL-1β and IL-18 [36].

Ceramides, another lipid species elevated in obesity, have also been shown to activate NLRP3. In cultured macrophages and adipose explants from obese mice, ceramides trigger caspase-1 activation, exacerbating inflammation and perpetuating metabolic imbalance [37].

Hyperglycemia, a defining feature of T2DM, also serves as a potent stimulus for NLRP3 activation. High glucose activates PKCα, which in turn stimulates NF-κB signaling through the phosphorylation of p38 MAPK and ERK1/2, promoting IL-1β transcription [38]. In parallel, glucose increases ROS production, which provides the second signal required for inflammasome assembly and cytokine maturation [39,40]. TXNIP, a key mediator of glucose-induced oxidative stress, is also upregulated under hyperglycemic conditions and enhances NLRP3 activation [41]. TXNIP regulates a range of metabolic processes, including β-cell function, peripheral glucose uptake, hepatic glucose production, and adipogenesis [41]. Animal studies show that TXNIP overexpression leads to β-cell apoptosis and impaired insulin sensitivity, while TXNIP deficiency protects against diet-induced insulin resistance and T2DM [41].

It is well-established that patients with diabetes often present with elevated uric acid levels, and recent studies highlight a mechanistic link between hyperuricemia and inflammation via NLRP3 inflammasome activation. Uric acid, particularly in crystalline form, acts as a DAMP that is capable of activating the NLRP3 inflammasome through pathways involving oxidative stress, mitochondrial dysfunction, and potassium efflux [42]. This activation leads to the release of IL-1β and IL-18, promoting inflammation.

In addition to its role in metabolism, the NLRP3 inflammasome also exerts important regulatory effects on gut microbiota composition. The intestinal microbiome plays a critical role in maintaining immune, metabolic, and barrier functions. Dysbiosis, or microbial imbalance, is increasingly linked to chronic inflammatory diseases, including T2DM. Mouse models have shown that the absence of inflammasome components results in increased susceptibility to colitis, tumorigenesis, and microbiota disruption [43,44]. NLRP3 contributes to maintaining intestinal epithelial integrity and coordinating host defense mechanisms, although the precise molecular mechanisms by which microbiota activate inflammasomes remain to be fully elucidated [43,44].

Together, these findings position the NLRP3 inflammasome as a central mediator of metabolic inflammation and a key contributor to the pathophysiology of T2DM. Its activation by nutrient-derived signals—including fatty acids, ceramides, and glucose—leads to a cascade of inflammatory responses that drive insulin resistance, β-cell dysfunction, and systemic metabolic derangement. The interplay between metabolic stress, innate immune activation, and gut microbial homeostasis highlights the broad pathogenic role of NLRP3 inflammasome in T2DM and related disorders.

## 5. NLRP3 Inflammasome and Diabetic Macrovascular Disease

Beyond its role in metabolic dysfunction, emerging evidence suggests that NLRP3 activation may also contribute to the development of macrovascular complications in T2DM. Chronic inflammasome activation not only disrupts cellular metabolism, but also exerts deleterious effects on vascular structure and function, linking innate immune activation with endothelial dysfunction, atherosclerosis, and cardiovascular disease (CVD) (Table 2).

Endothelial dysfunction is widely considered to be an early and pivotal event in the activation of the NLRP3 inflammasome in T2DM [57]. Endothelial cells, forming a single-cell lining of the vascular lumen, are responsible for a range of critical physiological functions. These include facilitating the transport of nutrients such as glucose, as well as hormones and macromolecules, from the bloodstream into surrounding tissues. They are also central to maintaining vascular integrity, regulating permeability, leukocyte trafficking, thrombus formation, fibrinolysis, and angiogenesis [57]. Endothelial cells secrete vasoactive substances to modulate vascular tone: vasoconstrictors such as endothelin-1 (ET-1) and thromboxane A2, and vasodilators including nitric oxide (NO), prostacyclin, and endothelium-derived hyperpolarizing factor [57]. As the interface between the bloodstream and tissues, they are fundamental to immune surveillance, acting through PRR to detect both PAMPs and DAMPs. Under pathological conditions, endothelial dysfunction is marked by impaired vasodilation, increased pro-inflammatory and pro-thrombotic activity, disrupted barrier function, and elevated oxidative stress.

In the context of DM, endothelial dysfunction emerges early and plays a key role in the development of hyperglycemia-associated vascular complications such as atherosclerosis. This is driven by a complex interplay involving oxidative stress, altered signaling pathways, mitochondrial dysfunction, and chronic low-grade inflammation. The loss of endothelial integrity facilitates vascular inflammation and permeability, ultimately contributing to diabetic vasculopathy. Accordingly, macrovascular complications are a leading cause of disability and mortality in individuals with T2DM [58,59].

T2DM is a major risk factor for CVD, increasing the risk of myocardial infarction and stroke two- to three-fold, predominantly due to accelerated atherogenesis. The NLRP3 inflammasome is now well recognized for its role in initiating and perpetuating vascular inflammation [60]. Atherosclerotic plaques demonstrate elevated mRNA and protein expression of NLRP3, ASC, caspase-1, IL-1β, and IL-18 in macrophages, foam cells, and endothelial cells [61]. IL-1β and IL-18 in particular have been strongly linked to plaque formation and instability [61].

NLRP3 inflammasome activation has been shown to occur in response to intracellular cholesterol crystals during atherogenesis. In vitro work by Duewell et al. revealed that cholesterol crystals are internalized by phagocytes, triggering NLRP3 inflammasome assembly via phagolysosomal membrane damage—highlighting cholesterol as both a metabolic and immunologic mediator in CVD [53].

A landmark study by Lee et al. established a direct connection between the NLRP3 inflammasome and diabetes-related CVD [47]. Monocytes from newly diagnosed treatment-naïve T2DM patients exhibited elevated expression of NLRP3 and ASC, as well as increased basal and DAMP-induced inflammasome activation. These patients also had significantly higher circulating levels of IL-1β and IL-18 compared to healthy controls. Notably, in vitro NLRP3 knockdown in monocytes from T2DM patients completely abrogated IL-1β and IL-18 secretion in response to metabolic DAMPs.

Consistent findings have been observed in preclinical models. In a rat model of T2DM, excessive NLRP3 inflammasome activation and pyroptosis were closely associated with pathological cardiac remodeling—effects that were significantly reversed following NLRP3 silencing [48]. Experimental studies using in vitro and in vivo models of atherosclerosis have further elucidated the role of NLRP3 inflammasome in diabetic macrovascular disease. In human umbilical vein endothelial cells (HUVECs) and in diabetic mouse atherosclerotic lesions, hyperglycemia was associated with the increased expression of endothelial adhesion molecules—a process that was significantly attenuated by NLRP3 knockdown or IL-1 receptor antagonism [49].

In addition to glucose-induced toxicity, the dysregulated lipid metabolism in diabetic vasculature may also contribute to NLRP3 inflammasome activation. In a porcine model of T2DM and atherosclerosis, increased expression of sterol regulatory element-binding protein (SREBP)-1 in the aorta correlated with elevated NLRP3 inflammasome components [52]. Immunostaining localized SREBP-1 expression to macrophages and endothelial cells in early-stage lesions (fatty streaks) and advanced plaques—findings mirrored in human aortic tissue samples from individuals with T2DM and atherosclerosis [52].

Beyond metabolic triggers, biomechanical forces have also been implicated in modulating NLRP3 inflammasome activity. Hemodynamic stress, particularly oscillatory shear stress, has been identified as a regulator of inflammasome activation in endothelial cells. This occurs via the downregulation of Kruppel-like factor 2 and the subsequent suppression of forkhead box P1, a transcription factor with anti-inflammatory functions [62]. Both human and murine atherosclerotic coronary arteries show reduced Foxp1 expression in areas of disturbed flow [62].

As previously discussed, a bidirectional relationship exists between impaired glucose metabolism and increased stroke risk. Insulin resistance not only serves as a precursor to T2DM, but is also an independent predictor of cardiovascular and cerebrovascular events [63]. Given this association, therapeutic targeting of the NLRP3 inflammasome presents a promising strategy for managing cerebrovascular ischemic disease in the context of insulin resistance.

In the setting of PAD, as shown in a study by Cai et al., diabetic PAD patients demonstrated markedly increased expression of NLRP3, ASC, and caspase-1 in affected arterial tissue compared to diabetic patients without PAD [64]. These findings suggest a role for NLRP3 inflammasome in the pathophysiological progression of diabetic PAD; however, the exact mechanisms and extent of its contribution remain to be fully clarified.

Heart failure is increasingly associated with chronic inflammation, with the NLRP3 inflammasome emerging as a key contributor. Activation of NLRP3 in cardiomyocytes and macrophages leads to caspase-1 activation, pyroptosis, and the release of IL-1β and IL-18, promoting myocardial inflammation, fibrosis, and adverse remodeling [65]. In pressure-overload and diabetic models, NLRP3 inhibition attenuates cardiac hypertrophy and improves function [65]. Pyroptosis, mediated by GSDMD, exacerbates cardiomyocyte loss and facilitates inflammatory spread to cardiac fibroblasts [65].

Despite growing interest, research into the molecular underpinnings of NLRP3 inflammasome in macrovascular complications beyond CVD has been relatively limited. The pathophysiological roles of the inflammasome in diabetic cerebrovascular disease and PAD are less well characterized in the literature, and additional mechanistic and translational studies are needed to bridge this gap.

Apart from CVD, the NLRP3 inflammasome is an important regulator of renal damage, across both acute and chronic renal pathologies. Upon activation in immune and non-immune renal cells, such as macrophages, tubular epithelial cells, and mesangial cells, the NLRP3 inflammasome triggers the maturation and release of pro-inflammatory cytokines IL-1β and IL-18, fueling inflammatory responses and cell death via pyroptosis [66]. In acute kidney injury (AKI), various insults—including contrast agents, sepsis, rhabdomyolysis, and chemotherapeutic drugs—stimulate NLRP3 activation through danger signals like ATP and reactive oxygen species, leading to tubular cell necrosis and inflammation. In chronic kidney disease (CKD), persistent NLRP3 activation drives renal fibrosis through the Transforming growth factor-β1/Smad signaling pathway and epithelial–mesenchymal transition, contributing to the progression of conditions such as diabetic nephropathy, crystal nephropathy, lupus nephritis, obesity-related fibrosis, and hypertension-induced damage. Therefore, the NLRP3 inflammasome acts as a key molecular hub linking inflammatory stimuli to kidney injury and fibrotic remodeling.

## 6. NLRP3 Inflammasome-Targeted Pharmacotherapy

**Figure 1:**
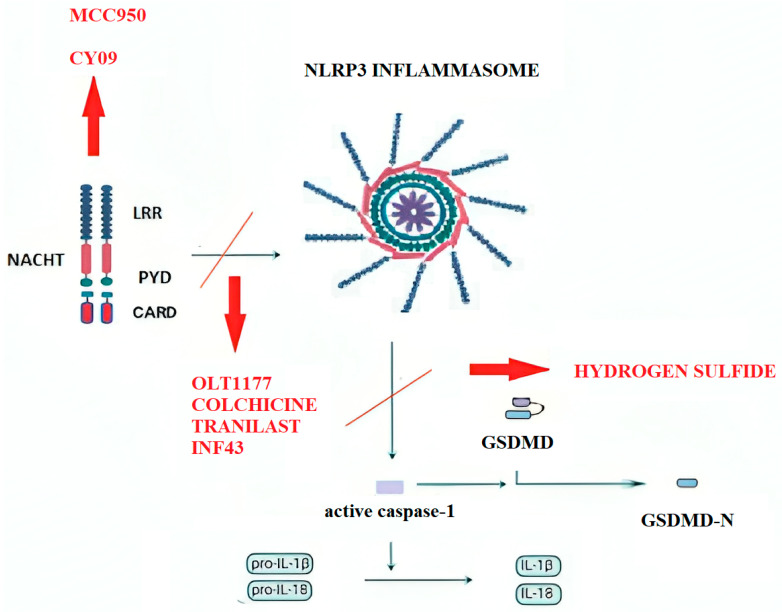
NLRP3 inflammasome inhibitors tested in the cardiovascular system and their site of action.

The well-established connection between the NLRP3 inflammasome and inflammatory, metabolic, and CVDs has driven substantial scientific interest in identifying pharmacological agents that target its activation (Figure 1, Table 3). Given its critical role in the pathogenesis of T2DM and CVD, NLRP3 has emerged as an attractive therapeutic target, prompting the development of several inhibitors that directly or indirectly modulate its signaling. Many of these agents are currently undergoing preclinical evaluation, with a few progressing to clinical trials. By interfering with NLRP3-mediated signaling at various stages—priming, activation, oligomerization, or downstream cytokine release—these compounds offer promising avenues for mitigating insulin resistance and inflammation in cardio-metabolic diseases.

### 6.1. MCC950

MCC950 (CP-456,773) is one of the most well-characterized selective NLRP3 inhibitors. It binds directly to the NACHT domain of NLRP3, inhibiting ATP hydrolysis and ASC oligomerization, thereby suppressing IL-1β release [68,83]. It has demonstrated efficacy in both canonical and non-canonical inflammasome activation without affecting other inflammasome complexes [102]. In various mouse models of diabetes-associated atherosclerosis and heart disease, MCC950 reduced plaque burden, improved vascular function, and attenuated myocardial remodeling. Despite its promising profile, clinical development was halted due to hepatotoxicity and renal inflammation.

### 6.2. Glyburide Derivatives

Glyburide, an antidiabetic sulfonylurea, was the first compound shown to selectively inhibit NLRP3 [83]. However, the high doses required for its anti-inflammatory effect limited its clinical utility [8]. Derivatives such as 16673-34-0 and JC-124, designed to exclude insulinotropic properties, showed significant cardioprotective effects in models of myocardial ischemia–reperfusion and doxorubicin-induced cardiotoxicity [14,83]. Their precise mechanism appears to involve the inhibition of NLRP3–ASC interaction and conformational changes in NLRP3, although further validation is needed [103].

### 6.3. Bay 11-7082

Bay 11-7082 is an IKKβ inhibitor that also exerts selective NLRP3 inhibition by targeting cysteine residues in the ATPase domain [73]. In myocardial ischemia–reperfusion models, it reduced leukocyte infiltration and infarct size [74]. However, due to its dual activity on NF-κB signaling, the exact mechanism behind its cardiovascular benefits remains unclear [14].

### 6.4. OLT1177

OLT1177 is a small, orally bioavailable molecule that disrupts NLRP3–ASC interaction and inhibits ATPase activity [75]. It selectively blocks IL-1β and IL-18 release. In myocardial ischemia models, OLT1177 significantly reduced infarct size and improved cardiac function [14,76]. In a Phase 1B study in patients with HFrEF, it was well tolerated and was associated with improved ejection fraction and exercise performance [77].

### 6.5. Colchicine

Colchicine, traditionally used for gout and pericarditis, interferes with NLRP3 assembly by disrupting microtubule dynamics and cellular trafficking [79]. It prevents P2X7-mediated pore formation and inhibits inflammasome oligomerization [80]. In murine myocardial infarction models, colchicine reduced inflammation and preserved cardiac function [81]. Large clinical trials such as COLCOT and LoDoCo demonstrated that low-dose colchicine reduces cardiovascular events and high-sensitivity C reactive protein levels in patients with recent myocardial infarction or stable coronary artery disease, supporting its potential use as a repurposed anti-inflammatory therapy [104].

### 6.6. CY-09

CY-09 is a small-molecule inhibitor that binds the ATP-binding site on NLRP3′s NACHT domain, effectively preventing its activation. In murine models of diabetic stroke and cardiac dysfunction, CY-09 improved outcomes by limiting inflammasome-driven inflammation [82]. It does not affect CFTR [12], a known off-target of related molecules, enhancing its safety profile.

### 6.7. Tranilast

Tranilast, originally developed for allergic conditions, directly binds the NACHT domain of NLRP3, preventing its oligomerization and ATPase activity without affecting upstream signals. In murine atherosclerosis models, it suppressed inflammasome assembly, enhanced NLRP3 ubiquitination, and limited plaque progression [71]. Clinical data suggest that Tranilast is well tolerated even at high doses, positioning it as a promising candidate for NLRP3-targeted therapy.

### 6.8. INF4E

INF4E is a synthetic compound that inhibits NLRP3 ATPase activity and caspase-1 activation [85]. In myocardial ischemia models, INF4E reduced infarct size, preserved cardiac function, and enhanced mitochondrial integrity [86]. However, its cytotoxicity prompted the development of modified derivatives such as INF58, which may offer improved safety, although their cardioprotective effects remain to be tested [83].

### 6.9. Hydrogen Sulfide Donors

Hydrogen sulfide (H_2_S) is an endogenous gasotransmitter with anti-inflammatory and cardioprotective properties. Donors such as Na_2_S and NaHS have been shown to reduce infarct size in myocardial ischemia–reperfusion models, partly by suppressing NLRP3-dependent caspase-1 activation [83,87]. H_2_S also appears to inhibit both the priming and activation phases of inflammasome signaling, and has been shown to mitigate NF-κB pathway activity in various experimental models.

## 7. Off-Target Modulation of the NLRP3 Inflammasome by Conventional Drugs and Natural Compounds

While numerous investigational agents have been designed to specifically inhibit the NLRP3 inflammasome, various conventional anti-diabetic, cardiovascular, and natural compounds have demonstrated off-target modulatory effects on this pathway. These effects, although not initially intended, offer promising insights into the broader therapeutic potential of widely used medications in mitigating the inflammation-driven complications of T2DM and CVD.

### 7.1. Diabetic Medications and NLRP3 Modulation

Several standard anti-diabetic drugs have been shown to attenuate inflammation [105] and NLRP3 inflammasome activity specifically, thereby offering benefits beyond glycemic control. Metformin, the most widely used first-line agent for T2DM, inhibits caspase-1 and IL-1β production while suppressing cell pyroptosis through AMPK/mTOR signaling, offering cardioprotective effects, particularly against ischemia–reperfusion injury [8,47].

SGLT2 inhibitors, such as dapagliflozin and empagliflozin, exhibit potent anti-inflammatory properties. Dapagliflozin reduces NLRP3 activation and improves cardiac function via AMPK/mTOR pathway modulation [89]. Empagliflozin, even in non-diabetic heart failure models, suppresses inflammasome activity and reduces intracellular calcium levels [88].

DPP4 inhibitors, including saxagliptin, exert protective effects against diabetic cardiomyopathy by restricting NLRP3 activation [8]. Similarly, pioglitazone, a thiazolidinedione, inhibits the NF-κB pathway, reduces ROS production, and has been shown to alleviate renal damage [90]. Acarbose, an α-glucosidase inhibitor, improves endothelial function by suppressing NOX4-dependent superoxide production and inhibiting NLRP3 activation in diabetic models [91].

### 7.2. Other Pharmaceutical Agents

Several cardiovascular and metabolic medications not originally developed for inflammasome targeting also exhibit NLRP3-modulating properties. Eplerenone, a potassium-sparing diuretic, reduces ROS generation and NF-κB phosphorylation while blocking transcription of NLRP3 components [92]. Verapamil, a calcium channel blocker, has been shown to suppress pathological neoangiogenesis and NLRP3 inflammasome activation in diabetic models [8].

Fenofibrate, a PPARα agonist used in managing hypertriglyceridemia, also appears to attenuate diabetic retinopathy, likely through inflammasome suppression, although this mechanism is not yet officially recognized [93]. Similarly, β-hydroxybutyrate, a ketone body produced during fasting or ketogenic diets, inhibits inflammasome activation by reducing K^+^ efflux and ASC oligomerization [95]. Its systemic anti-inflammatory effects, particularly in adipose tissue, warrant further exploration.

Statins, primarily used for lipid lowering, possess significant anti-inflammatory and immunomodulatory properties. Atorvastatin and simvastatin have demonstrated efficacy in reducing NLRP3 expression, attenuating cardiac fibrosis, and improving cardiac function in murine models, largely through TXNIP pathway inhibition [8,94].

### 7.3. Natural Compounds and Derivatives

An increasing number of natural substances have shown potential as NLRP3 inhibitors. Resveratrol, a polyphenol found in red grapes and peanuts, mimics metformin in activating AMPK and inhibits NLRP3 through reduced ER stress and mitochondrial fission [96,98]. In diabetic mice, resveratrol supplementation significantly reduced adipose inflammation and dysfunction [97].

Berberine, an alkaloid present in medicinal herbs, enhances autophagy, reduces ROS, and inhibits NLRP3 through AMPK activation. Its administration in HFD-fed mice improved glucose tolerance and insulin sensitivity [99]. Parthenolide, an alkylating agent derived from medicinal plants, disrupts NLRP3 ATPase activity, although its non-selective action also inhibits NLRP1, NLRC4, and caspase-1, raising concerns over potential off-target effects [17,96].

Melatonin, known for regulating circadian rhythms, downregulates NF-κB signaling and suppresses NLRP3, IL-1β, and pyroptosis markers in HFD-fed mice [100]. Additionally, glycyrrhizin (GL) and isoliquiritigenin (ILG), compounds from the Glycyrrhiza plant, inhibit TLR4 signaling and ASC oligomerization, effectively reducing both the priming and activation phases of inflammasome signaling [96]. ILG has been shown to suppress IL-1β production and adipose inflammation in murine models [101].

Together, these findings highlight the NLRP3 inflammasome as a shared mechanistic target among diverse therapeutic classes. While further research is needed to delineate dosage, specificity, and long-term effects, the off-target inhibition of NLRP3 by conventional and natural agents underscores the translational potential of repurposed or adjunctive therapies in treating inflammation-driven complications in T2DM and CVD.

## 8. Conclusions

The NLRP3 inflammasome plays a central role in the pathogenesis of T2DM and its macrovascular complications by linking metabolic stress to chronic inflammation. Its activation by hyperglycemia, saturated fatty acids, ceramides, and other endogenous danger signals leads to the secretion of IL-1β and IL-18, promoting insulin resistance, endothelial dysfunction, and atherosclerotic progression. Increasing evidence from both experimental and clinical studies supports the inflammasome’s contribution to diabetic cardiomyopathy, cerebrovascular disease, and PAD. Importantly, the discovery of selective NLRP3 inhibitors and the off-target anti-inflammatory effects of established diabetic and cardiovascular therapies highlight the inflammasome as being a promising target for therapeutic intervention. While further clinical validation is required, targeting the NLRP3 inflammasome may represent a transformative strategy for attenuating the inflammatory burden in T2DM and improving long-term cardiovascular outcomes. Continued exploration of NLRP3 modulation—both through novel agents and repurposed compounds—holds great potential for advancing personalized treatment in cardio-metabolic disease.

## Figures and Tables

**Table 1:** Summary of studies indicating a correlation between NRLP3 inflammasome and T2DM.

Authors	Year	Population	Study	Findings
Esser et al. [25]	2013	Human participants with different obesity phenotypes	Cross-sectional observational study	Increased expression of NLRP3 and IL1B in visceral adipose tissue from metabolically unhealthy obese patients
Yin et al. [26]	2014	Postmenopausal women, both lean and obese, undergoing elective abdominal surgery	Cross-sectional observational study	Genes associated with the NOD-like receptor pathway, including the NLRP3, were upregulated in adipocytes from obese individuals
Wang et al. [27]	2015	db/db mice	Pre-clinical experimental study (with in vivo and vitro methodologies)	NLRP3 and Caspase-1 expressions were increased in epididymal fat from db/db mice
Finucane et al. [28]	2015	C57BL/6 mice	Pre-clinical experimental study	NLRP3, Caspase-1, and IL1B expressions in adipose tissue were higher in mice treated for 6 months with a saturated fatty acid HFD in comparison with mice fed with a monounsaturated fatty acid HFD
Bitto et al. [29]	2014	db/db mice	Pre-clinical experimental study	NLRP3, ASC, caspase-1, IL-18, and IL-1 are upregulated during wound healing in animal models of T2DM in comparison with healthy animals
Coll et al. [30]	2019	Mouse bone marrow-derived macrophages and human monocyte-derived macrophages	Pre-clinical experimental study	MCC950, which inhibits the NLRP3 inflammasome, can be applied as a potential anti-inflammatory therapy in T2DM
Henriksbo et al. [31]	2014	ob/ob mice, 3T3-L1 adipocytes (murine adipocyte cell line)	Pre-clinical experimental study (with in vivo and vitro methodologies)	Fluvastatin provokes inflammation and insulin resistance in adipose tissue via the upregulation of NLRP3, which is consistent with the increased expression of NLRP3 in inflamed adipose tissues of T2DM patients
Kim et al. [32]	2016	Murine macrophage cell lines(iJ774) and bone marrow-derived macrophages	Pre-clinical experimental study (with in vivo and vitro methodologies)	NLRP3 can be suppressed by γ-tocotrienol, delaying the progression of T2DM

**Table 2:** Summary of studies correlating NRLP3 inflammasome with macrovascular disease in T2DM.

Authors	Year	Population	Study	Findings
Ridker et al. CANTOS trial [45]	2017	Patients with history of myocardial infarction and elevated hsCRP levels	Randomized, double-blind, placebo controlled, multicenter clinical trial	In total, 150 mg of Canakinumab significantly reduced cardiovascular death, providing the first definitive clinical evidence that reducing inflammation can lower CVD event risk
Yin Jin et al. [46]	2022	ApoE^-/–^ mice	Pre-clinical experimental study	Targeting caspase-1 and the NLRP3 assembly may offer therapeutic potential in atherosclerotic cardiovascular diseases.
Lee et al. [47]	2013	Patients with untreated T2DM	Comparative experimental study	Increased expression of the inflammasome components NLRP3 and ASC was found in monocytes from newly identified, untreated type 2 DM subjects
Luo et al. [48]	2014	HFD and STZ induced rat models	Pre-clinical experimental study	Diabetic rats showed severe metabolic disorder, cardiac inflammation, cell death, disorganized ultrastructure, fibrosis, and excessive activation of NLRP3
Wan et al. [49]	2019	Humans and ApoE^-/–^ mice	Pre-clinical experimental study (with in vivo and vitro methodologies)	NLRP3 was involved in hyperglycemia-induced endothelial inflammation, both in vitro and in vivo
Xiao-Xue Li et al. [50]	2019	Diabetic rats	Pre-clinical experimental study	High glucose induced the assembly and activation of NLRP3 inflammasome in endothelial cells
Feng et al. [39]	2016	Rat glomerular mesangial cells	Pre-clinical experimental study	High glucose levels and LPS exposure prime the NRLP3 inflammasome in mesangial cells through the ROS/TXNIP signaling pathway, leading to diabetic nephropathy
Sun et al. [51]	2019	STZ-induced diabetic rat model	Pre-clinical experimental study	Suppression of TXNIP/NLRP3 activation ameliorates diabetic peripheral neuropathy
Yu Li et al. [52]	2013	Porcine model of atherosclerosis and DM	Pre-clinical experimental in vivo study	In vivo evidence that the dysregulation of SIRT1-AMPK-SREBP and stimulation of NLRP3 inflammasome may contribute to vascular lipid deposition and inflammation in atherosclerosis
Duewell et al. [53]	2010	Mice deficient in components of the NLRP3 inflammasome	Pre-clinical experimental study (with in vivo and vitro methodologies)	Crystalline cholesterol acts as an endogenous danger signal and its deposition in arteries or elsewhere is an early cause rather than a late consequence of NLRP3 activation and inflammation
Kirii et al. [54]	2003	apoE^-/–^ and IL-1β^-/–^ mice	Pre-clinical experimental in vivo study	IL-1β deficiency significantly reduced atherosclerotic lesion size in the aorta, suggesting that IL-1β promotes atherogenesis through both immune cell recruitment and endothelial activation
Qian An et al. [55]	2017	STZ-induced diabetic rats	Pre-clinical experimental in vivo study	Suppression of the NLRP3 inflammasome pathway via oleanolic acid attenuates carotid artery injury in diabetic rats
Song et al. [56]	2015	Cultured endothelial cells	Experimental in vitro cellular study	Inhibition of ER stress-associated TXNIP/NLRP3 inflammasome activation in endothelial cells improves endothelial homeostasis

“-/–” means both copies (alleles) of the gene are knocked out (non-functional).

**Table 3:** Pharmacological approaches of NLRP3 inhibition.

	Drugs	Mechanism of Action	Studies	Findings	Status
NLRP3 inhibitors	MCC950 [67,68,69]	Non-covalent bonding to the NACHT domain	Many murine models (HFD, streptozotocin-induced ApoE^-/–^ mice, etc.) and Humans	Reduced atherosclerotic plaque development, decreased the expression of adhesion molecules within the plaque, and lowered the number of macrophages present in the plaque	Clinical development was discontinued due to excessive renal inflammation and hepatic toxicity
	Glyburide [70,71,72]	Inhibition of ATP-dependent potassium channels	Murine and humans models	Suppressed cardiac caspase-1 activity and minimized infarct size in mice undergoing myocardial ischemia followed by 24 h of reperfusion	Limited clinical use due to frequent hypoglycemia
	Bay 11-7082 [73,74]	NF-κΒ pathway inhibition	Myocardial ischemia–reperfusion murine models	Decreases leukocyte infiltration in the infarcted area and enhances cardiomyocyte survival, reducing infarct size	Pre-clinical studies
	OLT1177 [75,76,77]	Impairs ATPase activity of NLRP3	Animal models of myocardial ischemia–reperfusion	Dose-dependent reduction in infarct size, and also improved ventricular function in a model of permanent coronary artery occlusion	Pre-clinical studies
	Colchicine [78,79,80,81]	Interferes with the NLRP3 complex by disrupting microtubule action	Human studies (COLCOT, LoDoCo) and mouse models of permanent cardiac ligation	Decreased the infiltration of neutrophils and macrophages, as well as the mRNA expression of pro-inflammatory cytokines and NLRP3 inflammasome components 24 h after myocardial infarction	FDA-approved for inflammatory diseases
	CY-09 [12,82]	Inhibition of the NLRP3 complex by binding directly to the ATP-binding motif of the NACHT domain	Murine models of type 2 Diabetes Mellitus	Prevented cardiac dysfunction linked to diabetic ischemic stroke	Pre-clinical studies
	Tranilast [71,83,84]	Blocks the direct NLRP3-NLRP3 and NLRP3–ASC interaction	Mouse models of atherosclerosis and several animal models of hypertension, diabetic cardiomyopathy, and myocardial infarction	Enhanced NLRP3 ubiquitination, restricting NLRP3 inflammasome assembly and thereby reducing the initiation and progression of atherosclerotic plaques	Pre-clinical studies
	INF4E [85,86]	Inhibition of the NLRP3 ATPase activity	Murine models of myocardial ischemia	Reduced infarct size and improved left ventricular pressure	Clinical development was discontinued due to cytotoxic properties
	Hydrogen Sulfide [14,87]	Reduces NLRP3-dependent caspase-1 activation	Murine specimen undergoing ischemia–reperfusion injury	Diminished the IKKβ/NF-κB signaling pathway introducing cardioprotective properties in a hemorrhagic shock model	Pre-clinical studies
Anti-Diabetic Drugs	Metformin [8,47]	Activates AMPK that reduces ER stress and mitochondrial fission leading to inhibition of caspase-1	Studies in Monocyte-derived macrophages isolated from type 2 diabetic subjects	Protective properties against cell pyroptosis and myocardial ischemia–reperfusion injury by interfering with the AMPK/TOR signaling pathway	FDA-approved for Type-2 diabetes mellitus
	SGLT2 inhibitors [88,89]	Modulatory effects on the AMPK/TOR pathway	Eight-week-old BTBR and wild-type mice	Improved left ventricular end-systolic and end-diastolic volumes, as well as the left ventricular ejection fraction by modulating the AMPK/TOR pathway	FDA-approved for Type-2 diabetes mellitus and heart failure
	Pioglitazone [90]	Downregulation of NF-κB	apoE (^-/–^) mice	Reduced ROS releases and attenuated renal damage	FDA-approved for Type-2 diabetes mellitus
	Acarbose [91]	Inhibition of NOX4-depedant superoxide production	Rats with T2D	Enhanced endothelial function in the aorta of diabetic rats	FDA-approved for Type-2 diabetes mellitus
	Saxagliptin [8]	AMPK-dependent caspase-1 inhibition	Type 2 diabetic (BTBR ob/ob) and wild-type (WT) mice	Mitigate the advancement of diabetic cardiomyopathy	FDA-approved for Type-2 diabetes mellitus
Other pharmaceutical options	Eplerenone [92]	Inhibits phosphorylation of NF-κB and ROS production	C57BL/6 mice fed a high-fat diet (HFD)	Exhibited robust anti-inflammatory properties	FDA-approved drug for hypertension and heart failure
	Verapamil [8]	Inhibits the assembly of NLRP3, reduces the release of IL-1β	Mouse models with diabetic retinopathy	Attenuated pathological neo-angiogenesis	FDA-approved drug for hypertension and angina pectoris
	Fenofibrate [93]	Unidentified mechanism of NRLP3 inhibition	Mouse models with Diabetic Retinopathy	Attenuated retinal leukostasis, vascular leakage and the progression of DR	FDA-approved for hypertriglyceridemia
	Atorvastatin [8,94]	Inhibition of NLRP3 inflammasome via TXNIP	Murine models of diabetic cardiomyopathy	Ameliorated diastolic dysfunction and cardiac fibrosis	FDA-approved lipid-lowering agent
	β-hydroxybutyrate [95]	Abolishes K^+^ efflux and reduces ASC oligomerization and speck formation via unknown mechanism	Mouse models of ketogenic diet	Inhibited caspase-1 activation, and reduced neutrophil count and hyperglycemia	Pre-clinical studies
Natural Substances	Resveratrol [96,97,98]	Modulation of AMPK signaling pathway	Diabetic murine models	Restriction of inflammation and adipose dysfunction	Pre-clinical studies
	Berberine [99]	Enhances AMPK-dependent autophagy	HFD-fed murine models	Improved insulin sensitivity and glucose tolerance	
	Parthenolide [17,96]	Impairs ATPase activity of NLRP3, suppresses IκB kinase, and NF-κB	mouse ASC (polyclonal anti-mouse ASC), mouse NLRP3 (polyclonal anti-NLRP3 PYD), mouse caspase-1 p20 (monoclonal anti-mouse caspase-1 p20)	Exhibited anti-inflammatory properties via macrophage blockage	
	Melatonin [100]	suppresses NF-κB signaling by decreasing NF-κB and p65 protein levels in the cytoplasm and nucleus	HFD-fed murine models	Profound decrease in adipose tissue pyroptosis	
	Glycyrrhizin (GL) and Isoliquiritigenin (ILG) [96,101]	Inhibits mitogen-activated protein kinase (MAPK) activation	HFD-fed murine models	Diminished Il-1β production and adipose tissue inflammation	
